# Do all matches require the same effort? Influence of contextual factors on physical demands during official female handball competitions

**DOI:** 10.5114/biolsport.2024.136090

**Published:** 2024-04-25

**Authors:** Carlos García-Sánchez, Rafael Manuel Navarro, Daniel Mon-López, Raúl Nieto-Acevedo, Enrique Cañadas-García, Alfonso de la Rubia

**Affiliations:** 1 Deporte y Entrenamiento Research Group, Departamento de Deportes, Facultad de Ciencias de la Actividad Física y del Deporte (INEF), Universidad Politécnica de Madrid, C/Martín Fierro 7, 28040 Madrid, Spain; 2 Department of Sports Sciences, European University of Madrid; 28670 Villaviciosa de Odón, Spain

**Keywords:** tracking system external, load load monitoring local, positioning sytem, ultra-wideband technology

## Abstract

Understanding the influence of contextual factors on physical demands is essential to maximize performance in handball. The purpose of this study was to explore and compare the influence of contextual factors (halves of the match, level of the opponent, match outcome and player role) on external load during official matches in women’s handball. Twenty-two semi-professional female players from the Spanish 2^nd^ Division were monitored across 13 official home matches. Total distance covered (TDC), high-speed running distance (HSR), high-intensity breaking distance (HIBD), accelerations (ACC), decelerations (DEC) and PlayerLoad (PL) were collected in absolute and relative values (normalized by playing time) using a local positioning system (WIMU PRO, Realtrack Systems S.L., Almería, Spain). HSR, HSR/min and HIBD/min decreased during the second half (p < 0.05; small effects). Regarding the level of the opponent, high-level and middle-level teams elicited higher TDC/min, HIBD/min and PL/min than low-level teams (p < 0.05; small-moderate effects). Additionally, starter players exhibited higher absolute values of external load (TDC, HSR, HIBD, ACC, DEC and PL) compared to non-starters (p < 0.05; moderate-large effects). Match outcome did not affect the physical demands (p > 0.05). The study indicated that halves of the match, level of the opponent, and player role influenced external load experienced by players during official matches; specifically, starter players showed higher absolute values of external load compared to non-starters. This information should be considered in managing load and developing strategies to minimize fatigue and enhance performance during matches.

## INTRODUCTION

Success in handball is based on the ability to score more goals than the opponent at the end of two thirty-minute periods. Thus, determining and analysing performance factors is highly recommended to achieve victory [1]. Current literature indicates that handball performance is complex and multifactorial [1]. Consequently, player performance is the product of optimal integration and synergy of various attributes (e.g., health, technical and tactical skills, bioenergetics and neuromuscular abilities and capacities, anthropometric characteristics, and cognitive and psychosocial characteristics) [2, 3]. Therefore, individual performance is more strongly dependent on the interaction between a wide range of factors than on the maximum enhancement of one or two factors alone [3].

From a physical and physiological perspective, handball is considered an intermittent team sport characterized by long periods of low-intensity movements interrupted by short intervals of high-intensity actions with frequent and intense body contact against the opponents [2, 4]. During these high-intensity periods, players must perform different fundamental movement patterns (accelerations, decelerations, changes of direction, sprints and jumps) and handball- specific actions (passes, catches, throws and blocks) [2, 4]. Therefore, understanding the physical and mechanical demands during matches is essential to maximize performance, manage stress and recovery, and reduce injury risk [2, 4].

The investigation of the physical demands in handball has been developed by the analysis of the external and internal load [2, 5]. External load can be defined as the locomotor activities performed by players, which can be measured with standard units, such as kilograms, meters, seconds, velocity/speed, and power [6]. On the other hand, internal load refers to the individual psycho-physiological responses to the external load, which can be measured as heart rate, blood lactate, rating of perceived exertion, or biochemical and molecular responses [6]. In handball, the analysis of physical demands during official competitions has been done mainly through the external load assessment because internal load monitoring presents some practical limitations (e.g. blood sampling during matches) [7].

Traditionally, in handball, researchers have mostly used time–motion analysis to measure external load during training [8] and competition [9]. However, the emergence of new monitoring tools with a good level of validity [10, 11] and reliability [12], such as local positioning system (LPS) with ultra-wideband (UWB) technology or inertial measurement units (IMU) (e.g., accelerometer, magnetometer and gyroscope), has provided a precise and accurate understanding of the physical demands in male [13, 14] and female handball [15, 16]. Given these technological advances, García-Sánchez et al. [17] demonstrated that physical demands greatly depend on gender, competition level and playing positions. Nevertheless, as indicated by some researchers, few studies have examined the impact of the contextual factors on physical demands in handball [13, 14, 17].

Recent research carried out in other team sports (e.g. basketball, soccer or ice hockey) has revealed that physical demands are strongly influenced by contextual factors, such as match location (playing home or away) [18, 19], halves of the match (first half or second half) [19, 20], level of the opponent (high-level teams, intermediate- level teams or low-level teams) [18, 19, 20], match outcome (win, draw or loss) [18, 21], score differential (balanced or unbalanced matches) [22, 23] and player role (starter or non-starter) [24]. In handball, a recent systematic review [17] on external load during official elite competitions showed that only five studies have investigated the impact of halves of the match on external load outcomes. Some of these studies found that the time spent in high-intensity running during the match decreased in the second half [25–27]. Additionally, the total distance covered in the first ten minutes was slightly higher than that covered in the last ten minutes of the game [9]. Similarly, initial values of PlayerLoad/min declined throughout the halves [16]. Moreover, a new study conducted with four LIQUI-MOLY Handball-Bundesliga teams across three seasons showed that time spent at very-high speed and very-high metabolic power decreased from the first to second half [28].

However, evidence-based knowledge about the influence of contextual factors on external load during official matches in female handball is currently limited. Thus, the aim of the present study was to explore and compare the influence of contextual factors (i.e., halves of the match, level of the opponent, match outcome and player role) on physical demands during official matches in female handball players.

## MATERIALS AND METHODS

### Experimental approach to the problem

We conducted a retrospective observational design to determine the influence of contextual factors on physical demands in female handball players. LPS data collected correspond to the average values of 13 official home matches from the Spanish 2^nd^ Division during the 2021–2022 season (September-April). Players who participated for at least 1 min in each game were included in the study [15]. Goalkeepers were excluded from the analysis because running-based demands do not reflect their performance needs [29].

### Participants

Twenty-two female handball players from the same team (age: 20.5 ± 3.1 years; height: 168.0 ± 4.8 cm; and body mass: 66.1 ± 10.1 kg) participated voluntarily in this study. The players belong to the third tier of competition (highly trained or national level), according to the Participant Classification Framework provided by McKay et al. [30]. During the season, players typically performed four handball training sessions and two strength training sessions per week. Also, they participated regularly in one match per week. All players were informed of the study requirements and provided written informed consent prior to the start of the study. Additionally, all the ethical procedures used in this study were in accordance with the Declaration of Helsinki and were approved by the Ethics Committee of the European University of Madrid.

### Procedures and data analysis

The LPS system (WIMU PRO, Realtrack Systems SL, Almería, Spain) was installed on the official handball court where the team played their home matches according to the user manual and previous studies [13, 14]. Each player was fitted with a device on his back with an adjustable vest. Playing time was recorded only when the players were inside the court. Thereby, the manufacturer’s specific software (SPRO, version 958, Realtrack Systems SL, Almería, Spain) was used to calculate the perimeter of the court to determine the effective playing time. Thus, team time-outs (a maximum of three per team), periods when the game was interrupted (e.g., consultations between the referees or interruption to wipe the court) and the 2-minute suspension were omitted. During match play, all players were continuously monitored in real time. Subsequently, the individual LPS registers were exported to external memory and analysed using the manufacturer’s specific software. Finally, raw data were exported in Excel format and imported into the statistical software for statistical analysis.

### Contextual factors and external load variables

Four contextual factors were considered: (1) halves of the match (first half vs. second half); (2) level of the opponent – considering the final ranking of each team at the end of the competition [18] we established three tiers: ‘high-level teams’ (HLT) (1^st^ to 5^th^ place), ‘middle-level teams’ (MLT) (6^th^ to 10^th^ place), and ‘low-level teams’ (LLT) (11^th^ to 14^th^ place); (3) match outcome (win, draw or loss); (4) player role (starters vs. non-starters), considering the field players who start the match as starters and the rest of the players as non-starters. The number of matches and individual LPS registers for each contextual factor are shown in Table 1.

**TABLE 1:** Number of matches and individual LPS registers for each contextual factor.

Contextual factor	Variable	Matches	LPS registers
Halves of the match	First half	13	139
	Second half	13	133

Level of the opponent	High-level teams	4	50
	Middle-level teams	5	56
	Low-level teams	4	47

Match outcome	Win	9	107
	Loss	4	46

Player role	Starter	13	89
	Non-starter	13	64

In addition, a detailed description of the external load variables collected (distance, accelerometry and PlayerLoad) is shown in Table 2.

**TABLE 2:** Description of external load variables.

Variable	Unit	Description
Effective playing time	h:mm:ss	Time player spent on the court (within court limits)

Total distance covered (TDC)	m	Total distance covered by the player

Total distance covered/min (TDC/min)	m · min^−1^	Total distance covered per minute by the player

High speed running (HSR)	m	Total distance covered above 18.1 km/h

High speed running/min (HSR/min)	m · min^−1^	Total distance covered per minute above 18.1 km/h

High intensity break distance (HIBD)	m	Total distance covered with deceleration above 2 m · s^−2^

High intensity break distance/min (HIBD/min)	m · min^−1^	Total distance covered per minute with deceleration above 2 m · s^−2^

Accelerations (ACC)	count	Total number of accelerations performed by the player

Accelerations/min	count · min^−1^	Total number of accelerations per minute performed by the player

Decelerations (DEC)	count	Total number of decelerations performed by player

Decelerations/min	count · min^−1^	Total number of decelerations per minute performed by player

PlayerLoad (PL)	a.u.	Is a vector magnitude expressed as the square root of the sum of the squared instantaneous rates of change in acceleration in each one of the three planes divided by 100

PlayerLoad/min (PL/min)	a.u. · min^−1^	Is a vector magnitude expressed as the square root of the sum of the squared instantaneous rates of change in acceleration in each one of the three planes divided by 100 per minute

### Statistical analysis

Descriptive statistics are presented as means and standard deviations (M ± SD). The level of significance was set at *p* < 0.05. Before carrying out the analyses, the Kolmogorov-Smirnov test was performed to confirm data distribution normality and Levene’s test for equality of variances. Differences between halves of the match were determined by the independent T-test (parametric variables) or Mann-Whitney U test (non-parametric variables). Level of the opponent differences were determined by one-way analysis of variance (ANOVA) followed by Games–Howell or Tukey post hoc testing (parametric variables), or the Kruskal-Wallis test followed by the Dwass-Steel-Critchlow-Fligner test (non-parametric variables). Differences according to player role and match outcome were evaluated through mixed two-way ANOVA followed by the Bonferroni post-hoc test (starter vs. non starter as repeated measure, and win vs. loss as independent measure). Furthermore, partial etasquared (η*p*^2^) or epsilon-squared (ε^2^) was calculated for group effects with the following interpretation: > 0.01 *small,* > 0.06 *moderate,* and > 0.14 *large* [31]. For the post-hoc analysis, Cohen’s d (ES) was calculated and interpreted using Hopkins’ categorization criteria: *d* > 0.2 as *small, d* > 0.6 as *moderate d* > 1.2 as *large,* and *d* > 2.0 as *very large* [32]. Data analysis was performed using SPSS for Windows (Version 26, IBM Corp., Armonk, NY, USA).

## RESULTS

### Halves of the match

**FIG. 1:**
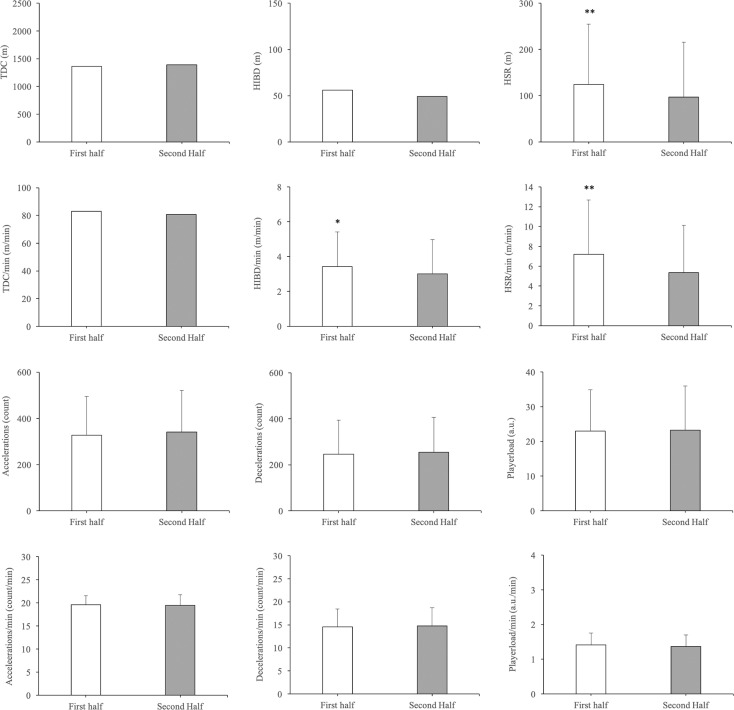
Influence of halves of the match on external load variables. Significance level between first and second half = *p < 0.05; **p < 0.01; ***p < 0.001.

There were significant differences between halves of the match with small effect sizes in HSR (*p* = 0.002, ES = 0.22), HSR/ minute (*p* = 0.001, ES = 0.36) and HIBD/minute (p = 0.046, ES = 0.20) (Figure 1). HSR and HSR/minute distance were slightly higher during the first (124.25 ± 130.41 m; 7.19 ± 5.47 m · min^−1^, respectively) than the second half (96.74 ± 118.99 m; 5.33 ± 4.78 m · min^−1^, respectively) (*p* < 0.01, ES: 0.22; *p* < 0.01, ES: 0.36). Also, HIBD/minute distance was slightly higher during the first (3.41 ± 1.99 m · min^−1^) than the second half (3.01 ± 1.95 m · min^−1^) (*p* < 0.05, ES: 0.20). No significant differences in the rest of the external load variables were found (*p* > 0.05).

### Level of the opponent

**FIG. 2:**
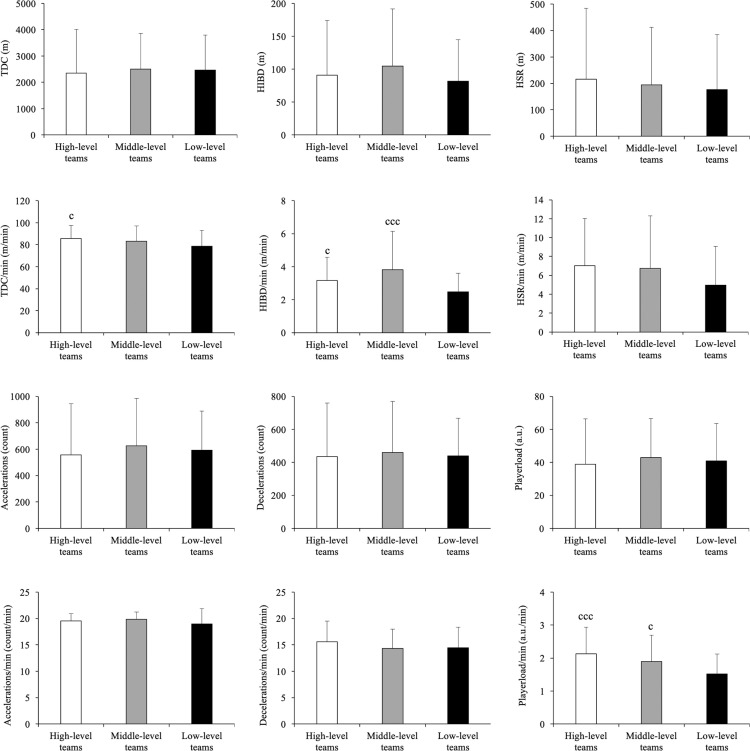
Influence of level of the opponent on external load variables. Significance level is indicated by the number of symbols: one symbol for *p* < 0.05, two for *p* < 0.01, and three for *p* < 0.001. ^a^ indicates significant differences vs. high-level teams, ^b^ indicates significant differences vs. middle-level teams and ^c^ indicates significant differences vs. low-level teams.

There were significant differences according to level of the opponent with a moderate to large effect size in TDC/min (*p* = 0.033, *ηp^2^* = 0.444), HIBD/min (*p* < 0.001, ε^2^ = 0.070) and PL/min (*p* < 0.001, ε^2^ = 0.116) (Figure 2). TDC/min was slightly higher in matches involving HLT (85.55 ± 12.25 m · min−1) compared to matches with LLT (78.48 ± 14.36 m · min^−1^) (*p* < 0.05, ES: 0.51). Also, HIBD/min distance was moderately higher in matches involving HLT (3.16 ± 1.41 m · min^−1^) and MLT (3.96 ± 2.38 m · min^−1^) compared to LLT (2.46 ± 1.13 m · min^−1^) (*p* < 0.05, ES = 0.39; *p* < 0.001, ES = 0.84, respectively). Additionally, matches involving HLT (2.13 ± 0.80 arbitrary units (a.u.) · min^−1^) and MLT (1.93 ± 0.82 a.u. · min^−1^) registered moderately more PL/min compared to LLT (1.51 ± 0.60 a.u. · min^−1^) (*p* < 0.001, ES = 0.81; *p* < 0.05, ES = 0.55, respectively).

### Player role and match outcome

**FIG. 3:**
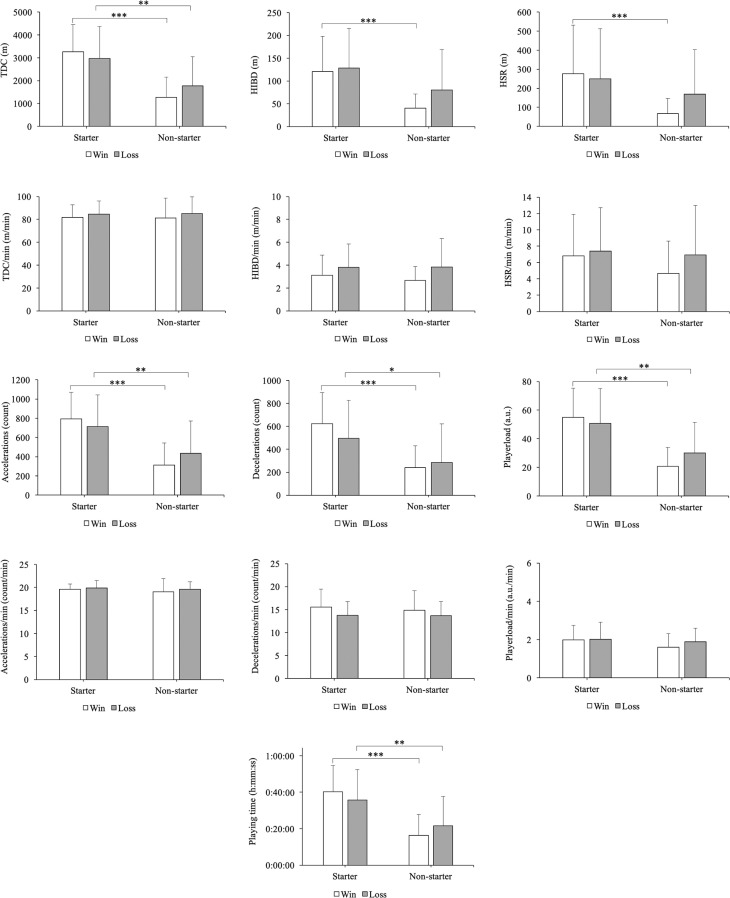
Influence of match outcome and player role on external load variables. Significance level is indicated by the number of symbols: one symbol for *p* < 0.05, two for *p* < 0.01, and three for *p* < 0.001. * indicates significant differences between starters and non-starters.

There were significant differences between starter and non-starter players with a moderate to large effect size in playing time (p < 0.001, η*p*^2^ = 0.270), TDC (p < 0.001, η*p*^2^ = 0.283), HSR *(p* < 0.001, η*p*^2^ = 0.085), HIBD (p < 0.001, η*p*^2^ = 0.146), ACC (p < 0.001, η*p*^2^ = 0.273), DEC (p < 0.001, η*p*^2^ = 0.242) and PL (p < 0.001, η*p*^2^ = 0.292) (Figure 3). In terms of player role, starters registered, with a moderate to large effect size, more playing time than non-starters in both winning (+24.1 min, *p* < 0.001, ES = 1.68) and losing conditions (+14.1 min, *p* = 0.007, ES = 0.98). Likewise, starters evidenced a moderate to large increase in TDC compared to non-starters in both winning (+1990.43 m, *p* < 0.001, ES = 1.71) and losing conditions (+1203.99 m, *p* = 0.004, ES = 1.04). In addition, starters performed, with a moderate to large effect size, more ACC, DEC and PL than non-starters in both winning (+479.75, *p* < 0.001, ES = 1.70; +382.83, *p* < 0.001, ES = 1.60; +34.27 a.u., *p* < 0.001, ES = 1.75, respectively) and losing conditions (+277.53 m, *p* = 0.007, ES = 0.98; +210.92, *p* = 0.021, ES = 0.88; +20.72 a.u., *p* < 0.05, ES = 1.06, respectively). Lastly, starters covered moderately more HSR and HIBD distance than non-starters in the win matches (+207.94 m; *p* < 0.001, ES = 0.96; +80.11 m, *p* < 0.001, ES = 1.13, respectively). In contrast, the comparative analysis according to match outcome (win vs. loss) dis not reveal significant differences in external load variables (p > 0.05). Furthermore, no significant interaction effect (player role vs. match outcome) was found in any external load variables (p > 0.05).

## DISCUSSION

The aim of this study was to explore and compare the external load measured during official matches according to halves of the match (first vs. second), level of the opponent (HLT vs. MLT vs. LLT), match outcome (win vs. loss) and player role (starter vs. non-starter) in semi-professional female handball. The main findings indicated that: (1) there were significant decreases from the first to the second half in high-intensity distance variables (HSR and HIBD); (2) HLT elicited higher TDC/min, HIBD/min and PL/min (moderate to large effect) than LLT; (3) starters exhibited higher absolute values of external load (moderate to large effect) compared to non-starters; (4) there were no significant differences in the external load between win and loss matches.

Regarding halves of the match, there was a significant decrease with small effect sizes in HSR, HSR/min and HIBD/min in the second half. In this regard, other studies carried out with elite male and female handball players have demonstrated that the time spent in high-intensity running [25–28] and the values of PL/min decreased in the second half [16]. Similar results were found in basketball, where high-intensity variables studied during the first quarter were higher than in all other quarters [20], and in soccer, where external load was higher during the first half than the second half [19]. There are many factors contributing to the decline in HSR, HSR/min and HIBD/min. First, it is well documented that repeated high-intensity actions (associated with intense eccentric contractions) and received multiple collisions and contacts during the match may produce fatigue, tissue damage and inflammatory responses, and impair neuromuscular performance [33, 34]. In addition to fatigue mechanisms, the decline in HSR, HSR/min and HIBD/min may be related to contextual factors such as score differential (balance vs. unbalance matches) [22, 23] or tactical strategies (e.g. different defensive systems or pacing strategies) [13]. Therefore, handball coaches should incorporate four types of interventions to minimize the occurrence of fatigue during the match: (1) increase player rotations to avoid excessive physiological load [9, 13, 15]; (2) improve basic (aerobic) and specific endurance to repeat high-intensity actions [1, 2]; (3) increase the capacity of muscles and tendons to attenuate efficiently high braking eccentric forces [34, 35]; (4) include half-time strategies (e.g. rehydrate, re-fuel, ergogenic aids, heat maintenance, rewarm up) to increase second-half performance [36].

Findings from the present study also showed that HLT elicited higher TDC/min, HIBD/min and PL/min than LLT. This difference may be explained by the faster play and the higher number of fast breaks performed by HLT compared to LLT [37, 38]. Similarly, previous studies with other modalities (e.g. soccer) have observed that the distance covered at high-intensity running was greater in matches against HLT [39, 40]. Consequently, players must be physically well prepared (i.e., strength and endurance) to play against HLT. Therefore, our findings suggest that handball coaches should design and implement three types of interventions against HLT: (1) develop a substitutions strategy to ensure that players perform at high intensity during the entire match [9, 13, 15]; (2) adapt or reduce training load days prior to the competition to avoid excessive fatigue and ensure optimal player readiness (micro-tapering) [41]; (3) incorporate some priming strategies to elicit a delayed potentiation (e.g. traditional strength exercises performed with low volume, high load and maximal intended velocity) [42, 43] or nutrition and supplementation practices (e.g. caffeine intake) [41] to enhance neuromuscular performance during a match.

Although unlimited substitutions are allowed in handball, our study revealed that starters accumulate more TDC, HSR, HIBD, ACC, DEC and PL than non-starters. Nevertheless, when these values were normalized according to the effective playing time (n · min^−1^), the differences in external load between starters and non-starters disappeared. It is difficult to compare the findings of the present study with previous research because there is a lack of scientific evidence comparing differences in external load between starters and non-starters in handball. However, we found similar results in soccer, where external load was higher in players from the starting lineups [44]. Thus, our results could be explained in part by the higher playing time for starters, due to the fact that there is a direct relationship between time on court and external load (in absolute values) accumulated by players. Several inferences can be drawn from our findings: (1) if we want to compare better the external load registered by starters and non-starters, we should use normalized values according to playing time; (2) the coach’s decisions (game plan and substitutions) have a direct impact on the external load that the players accumulated. Therefore, it could be expected that proper management of substitutions can contribute to a better load distribution among all players in order to minimize the occurrence of fatigue during the match, reduce injury risk, improve post-match recovery and increase player availability during match-congested schedules [3]. Also, as in other team sports (e.g. soccer and basketball), handball staff should consider the different absolute external load accumulated by starters and non-starters to better prescribe the training load across the microcycle [24, 45]. For example, practitioners should balance the chronic workload of non-starters who only play “junk minutes” [45] with compensatory training interventions during the microcycle.

Regarding match outcome, there are no significant differences in the external load between win and loss matches. These results are consistent with a previous study [46] confirming that there were no differences between winners and losers in the total distance covered and running pace during the matches. In the same line, a recent study conducted with four LIQUI-MOLY Handball-Bundesliga teams across three seasons reported that winning teams did not differ from drawing teams in terms of high-intensity running actions [28].

Some limitations should be considered when interpreting the current findings. First, external load was monitored only during thirteen official home matches. Accordingly, it should be noted that some variables could have influenced our results, such as match location (away matches were not monitored) and score differential (balance vs. unbalance matches). Second, a very particular sample (i.e., a Spanish 2^nd^ Division team) and not particularly large *(n* = 22) was used. Thirdly, our results did not differentiate playing position and attacking or defensive specialist players. Lastly, as it was an observational study, player rotations could not be controlled or influenced by researchers. Thus, coaches and practitioners should generalize and extrapolate the results with caution. In this regard, future research should include a larger number of teams and different levels of competition.

## CONCLUSIONS

The present study indicates that the physical demands of semiprofessional female players are influenced by halves of the match, level of the opponent and player role. However, match outcome does not affect the physical demands. Specifically, HSR, HSR/min and HIBD/min decreased during the second half. Regarding the level of the opponent, high-level teams elicited higher TDC/min, HIBD/min and PL/min than low-level teams. Additionally, starter players exhibited higher absolute values of external load compared to non-starters. Consequently, handball coaches should incorporate different interventions (e.g. player rotations) to minimize the occurrence of fatigue during the second halves of matches. Likewise, they should include several strategies (e.g. priming resistance training or micro-tapering) to enhance neuromuscular performance against high-level teams. Lastly, they should consider the different absolute external load experienced by starters and non-starters to better prescribe the training load across the microcycle.
